# Joint Association of Inflammatory‐Metabolic Indices With Risk of Incident Stroke Among Middle‐Aged and Older Chinese Adults

**DOI:** 10.1155/jobe/8894079

**Published:** 2026-09-28

**Authors:** Binbin Wang, Shuke Liu, Na Meng, Quan Li, Yan Chen, Xiaoxiao Peng, Haohao Wu, Shanwen Zhang, Gangzhen Qian

**Affiliations:** ^1^ Department of Cardiology, The Chenggong Hospital Affiliated to Xiamen University, Xiamen, Fujian, China, xmu.edu.cn; ^2^ Department of Cardiology, Guiqian International General Hospital, Guiyang, Guizhou, China; ^3^ Department of Orthopedics, The Chenggong Hospital Affiliated to Xiamen University, Xiamen, Fujian, China, xmu.edu.cn; ^4^ Lung Cancer Center, West China Hospital, Sichuan University, Chengdu, Sichuan, China, scu.edu.cn

**Keywords:** CHARLS, CTI–CVAI, CTI index, CVAI, incident stroke

## Abstract

**Aims:**

To investigate the independent and joint associations of the C‐reactive protein–triglyceride–glucose index (CTI) and the Chinese visceral adiposity index (CVAI) with incident stroke. Additionally, we evaluated whether a novel combined index, CTI–CVAI, outperforms the established TyG–CVAI in predicting stroke risk.

**Materials and Methods:**

This cohort study included 8267 stroke‐free participants from the China Health and Retirement Longitudinal Study (CHARLS). Cox proportional hazards models, Kaplan–Meier curves, and restricted cubic splines were utilized to analyze associations with incident stroke. Additive and multiplicative interactions between CTI and CVAI were examined. Predictive performance was assessed via receiver operating characteristic (ROC) analysis, comparing CTI–CVAI against TyG–CVAI using DeLong’s test and the Boruta algorithm for feature importance.

**Results:**

During a mean follow‐up of 8.37 years, 816 incident stroke cases were identified. Elevated CTI and CVAI were independently associated with increased stroke risk in a linear dose–response manner, without significant interaction. The novel CTI–CVAI index exhibited a robust association with stroke; participants in the highest tertile had a 2.14‐fold higher risk (HR 2.14, 95% CI 1.75–2.62) compared to the lowest. Crucially, CTI–CVAI demonstrated superior predictive accuracy (AUC 0.627) over the established TyG–CVAI index (*P* = 0.002), a finding supported by consistent results across subgroups.

**Conclusions:**

CTI and CVAI are independent risk factors for stroke, and their combination (CTI–CVAI) offers enhanced predictive value, outperforming the TyG–CVAI index. Monitoring CTI–CVAI may serve as a superior strategy for early risk stratification and targeted stroke prevention in middle‐aged and elderly populations.

## 1. Introduction

Stroke remains a leading global cause of death and disability, second only to ischemic heart disease in mortality and ranking third in disability‐adjusted life years [1]. Approximately 6.55 million stroke‐related fatalities and 101 million prevalent cases occurred worldwide in 2019 alone [2]. Beyond its high mortality rate, stroke imposes a substantial burden through physical disability, cognitive impairment, and chronic pain, severely compromising the quality of life for survivors [3–5]. Consequently, lowering stroke incidence and improving prognosis depend heavily on the early detection and management of modifiable risk factors.

Insulin resistance is a well‐established predictor of cerebrovascular pathology in both diabetic and nondiabetic populations, independently forecasting the onset and poor prognosis of stroke [6, 7]. As a reliable and accessible proxy for insulin resistance, the triglyceride–glucose (TyG) index has garnered significant attention [8]. Accumulating evidence suggests that both elevated baseline values and long‐term cumulative exposure to high TyG levels are potent drivers of stroke susceptibility [9, 10]. The prognostic utility of the TyG index can be further amplified when integrated with anthropometric parameters. For instance, a composite metric combining the TyG index and body mass index (TyG–BMI) outperforms the predictive value of either component alone [11]. Integrating the TyG index with the Chinese visceral adiposity index (CVAI) to specifically capture visceral fat accumulation has also exhibited superior performance in stroke risk stratification compared to other TyG‐derived metrics [12, 13].

The recently proposed C‐reactive protein–triglyceride–glucose (CTI) index innovatively combines the traditional TyG index with C‐reactive protein (CRP) [14]. By incorporating inflammatory data into a metabolic framework, this composite indicator mirrors the intricate interplay between insulin resistance and chronic systemic inflammation, offering a comprehensive assessment of cardiometabolic health [15]. Emerging evidence suggests that elevated CTI levels are linked to heightened stroke susceptibility and may surpass the traditional TyG index in predictive precision [16].

Despite these insights, the potential synergistic utility of integrating CTI with CVAI remains uninvestigated. We hypothesized that elevated CTI and CVAI levels are independently and jointly associated with an increased risk of incident stroke. We further hypothesized that their novel composite metric (CTI–CVAI) improves the identification of high‐risk individuals for early preventive intervention. Accordingly, this study aims to evaluate the prognostic value of CTI–CVAI and elucidate the associations of its individual components with stroke incidence.

## 2. Materials and Methods

### 2.1. Study Design and Population

Data were obtained from the China Health and Retirement Longitudinal Study (CHARLS), a nationwide prospective cohort of adults aged 45 years and older across 28 provinces. The initial recruitment in Wave 1 (2011–2012) served as the baseline for this study, providing the essential blood biomarker data required to calculate the CTI and CVAI indices. To maximize follow‐up duration and assess long‐term prognostic effects, outcome data were collected from subsequent surveys extending from Wave 2 (2013) through Wave 5 (2020). The study protocol received ethical clearance from the Peking University Institutional Review Board (approval number: IRB00001052‐11015), and all subjects provided written informed consent prior to enrollment.

Starting with the baseline cohort (*N* = 17,708), we applied a rigorous multistep filtration process to identify eligible participants. Exclusion criteria included age < 45 years or unrecorded age, incomplete data for computing CTI or CVAI, presence of extreme outliers in key exposure metrics, a pre‐existing diagnosis of stroke at baseline, and loss to follow‐up. A final analytical sample of 8267 individuals was retained. A comprehensive visualization of the inclusion and exclusion pathways is presented in Figure S1.

### 2.2. Exposure Assessment and Index Calculation

This study evaluated three continuous exposure variables and a derived categorical joint exposure. The CTI was calculated by integrating high‐sensitivity CRP with the TyG index based on established methodology [16]. The CVAI was computed using sex‐specific algorithms that incorporate age, BMI, waist circumference, triglycerides (TG), and high‐density lipoprotein cholesterol (HDL‐C) [17]. All lipid concentrations were standardized to mmol/L prior to calculation to ensure consistency with the original validation study [12]. Following previous approaches for constructing composite metrics, we generated the novel CTI–CVAI index by multiplying the individual CTI and CVAI values [13]. To further assess the cumulative risk profile, we dichotomized participants using the baseline median values of 8.67 for CTI and 93.42 for CVAI. This stratification yielded four distinct joint exposure categories: low CTI with low CVAI, high CTI with low CVAI, low CTI with high CVAI, and high CTI with high CVAI (detailed mathematical formulas for all indices are provided in Table S1).

### 2.3. Ascertainment of Incident Stroke

The primary endpoint was the first occurrence of stroke recorded during the follow‐up period from Wave 2 (2013) to Wave 5 (2020). Outcome status was determined through self‐reported physician diagnoses. Specifically, participants were classified as having an incident stroke if they affirmed receiving a medical diagnosis of the condition during the follow‐up interviews. This classification was further corroborated by data regarding ongoing stroke‐related treatments.

### 2.4. Assessment of Covariates

We extracted a broad array of baseline covariates to adjust for potential confounding. The analysis incorporated continuous age data alongside categorical variables defining sex as male or female, residence as urban or rural, and marital status as married versus unmarried, divorced, or widowed. Education was stratified based on high school completion, and lifestyle factors were classified by whether participants currently smoked or consumed alcohol. Chronic disease status was determined by triangulating survey responses with clinical data. Hypertension was identified in participants reporting a prior diagnosis, receiving antihypertensive therapy, or presenting with a systolic blood pressure of at least 130 mmHg or a diastolic blood pressure of at least 80 mmHg. Diabetes mellitus was defined by a history of diagnosis, use of glucose‐lowering agents, a fasting glucose level of 126 mg/dL or higher, or a glycated hemoglobin (HbA1c) level of 6.5% or greater. Key lipid parameters, including total cholesterol (TC) and low‐density lipoprotein cholesterol (LDL‐C), were also quantified.

### 2.5. Statistical Analysis

Baseline characteristics were summarized according to the tertiles of CTI, CVAI, and CTI–CVAI. Continuous variables were assessed for normality using the Kolmogorov–Smirnov test and presented as means ± standard deviations (SDs) or medians with interquartile ranges (IQR). Categorical variables were reported as frequencies and percentages. Group differences were evaluated using one‐way ANOVA or the Kruskal–Wallis test for continuous variables and the chi‐square test or Fisher’s exact test for categorical variables. Incidence rates were computed as the number of new stroke cases per 1000 person‐years. Stroke‐free survival probabilities were estimated using Kaplan–Meier curves and compared via the log‐rank test. The proportional hazards assumption was verified by testing the interaction between variables and the logarithm of time, with no violations observed (likelihood ratio test, *p* > 0.05). Cox proportional hazards regression models were constructed to estimate hazard ratios (HRs) and 95% confidence intervals (CIs) for the associations of the indices with incident stroke. Three models were fitted: Model 1 was unadjusted; Model 2 was adjusted for age, sex, education, marriage, and residence; and Model 3 was fully adjusted for variables in Model 2 plus smoking, drinking, diabetes, hypertension, TC, and LDL‐C.

Multiplicative and additive interaction analyses were performed to explore interactive effects between CTI and CVAI. Additive interaction was evaluated using the relative excess risk due to interaction (RERI), the attributable proportion (AP) due to interaction, and the synergy index (SI) [18]. Restricted cubic splines (RCS) with three knots were employed to visualize potential nonlinear dose–response relationships. Receiver operating characteristic (ROC) curve analysis was performed to assess diagnostic value. The area under the curve (AUC) was calculated, and DeLong’s test was specifically used to compare the predictive performance of CTI–CVAI against TyG–CVAI. The Boruta algorithm [19], a random forest‐based feature selection method, ranked the importance of key predictors. Subgroup analyses explored potential heterogeneity. The conservative Bonferroni method was applied in sensitivity analyses to adjust for multiple comparisons.

R software Version 4.4.0 was used for all statistical analyses. Statistical significance was defined as a two‐sided *p* value < 0.05.

## 3. Results

### 3.1. Baseline Characteristics of Participants

During a cumulative observation period of 70,164.98 person‐years (mean follow‐up: 8.37 years), we identified 816 incident stroke cases, yielding an incidence density of 11.63 per 1000 person‐years (95% CI: 10.85–12.46). Table 1 details the clinical and demographic characteristics of the study population (*N* = 8267) stratified by CTI–CVAI tertiles. The cohort’s mean age was 59.40 ± 9.23 years, and 53.5% were female. A progressive deterioration in cardiometabolic health was evident across ascending tertiles. Compared to the lowest tertile (T1), the highest tertile (T3) exhibited a clear high‐risk phenotype, characterized by a significantly larger burden of diabetes mellitus (25.3% vs. 7.7%) and hypertension (74.1% vs. 38.8%; all *p* < 0.001), alongside a higher prevalence of urban residence and advanced age. Regarding clinical and laboratory profiles, the T3 cohort displayed an adverse metabolic signature with elevated waist circumference, BMI, CRP, fasting blood glucose, HbA1c, and TC, alongside suppressed HDL‐C levels (all *p* < 0.001). Conversely, T1 contained a higher proportion of males and active smokers or drinkers. Stratification by individual components (CTI and CVAI) revealed similar age‐dependent and metabolic risk gradients (Tables S2 and S3). The composite CTI–CVAI index showed strong concordance with its constituent metrics and the TyG–CVAI comparator, all increasing significantly across tertiles (*p* < 0.001).

**TABLE 1:** Baseline characteristics of participants according to tertiles of the CTI–CVAI index.

Characteristics	Total (*n* = 8267)	Tertile 1 (*n* = 2756)	Tertile 2 (*n* = 2755)	Tertile 3 (*n* = 2756)	*p* value
Age (years)	59.40 ± 9.23	57.43 ± 8.70	59.49 ± 9.24	61.27 ± 9.34	< 0.001
Sex, *n* (%)					< 0.001
Female	4421 (53.5)	1225 (44.4)	1646 (59.7)	1550 (56.2)	
Male	3846 (46.5)	1531 (55.6)	1109 (40.3)	1206 (43.8)	
Education, *n* (%)					0.197
Below junior high school	7531 (91.1)	2518 (91.4)	2524 (91.6)	2489 (90.3)	
High school and above	736 (8.9)	238 (8.6)	231 (8.4)	267 (9.7)	
Marital status, *n* (%)					0.114
Married/cohabiting	6910 (83.6)	2324 (84.3)	2270 (82.4)	2316 (84.0)	
Other	1357 (16.4)	432 (15.7)	485 (17.6)	440 (16.0)	
Residence, *n* (%)					< 0.001
Urban	2695 (32.6)	689 (25.0)	852 (30.9)	1154 (41.9)	
Rural	5572 (67.4)	2067 (75.0)	1903 (69.1)	1602 (58.1)	
Smoking, *n* (%)	2512 (30.5)	1103 (40.1)	725 (26.4)	684 (24.9)	< 0.001
Drinking, *n* (%)	2723 (33.0)	1088 (39.5)	814 (29.6)	821 (29.8)	< 0.001
WC (cm)	85.13 ± 10.05	75.97 ± 5.69	84.52 ± 5.92	94.90 ± 7.47	< 0.001
BMI (kg/m^2^)	23.46 ± 3.86	20.57 ± 2.28	23.17 ± 2.69	26.65 ± 3.71	< 0.001
Diabetes, *n* (%)	1250 (15.1)	211 (7.7)	343 (12.5)	696 (25.3)	< 0.001
Hypertension, *n* (%)	4592 (55.7)	1065 (38.8)	1492 (54.3)	2035 (74.1)	< 0.001
CRP (mg/L)	1.03 (0.55, 2.15)	0.67 (0.40, 1.32)	0.96 (0.54, 1.97)	1.59 (0.89, 3.17)	< 0.001
FBG (mg/dL)	110.28 ± 36.26	101.60 ± 22.02	107.65 ± 33.25	121.58 ± 46.31	< 0.001
HbA1c (%)	5.27 ± 0.81	5.12 ± 0.59	5.21 ± 0.73	5.48 ± 1.01	< 0.001
TC (mg/dL)	194.01 ± 38.76	186.08 ± 35.50	193.09 ± 36.93	202.84 ± 41.73	< 0.001
HDL‐C (mg/dL)	51.34 ± 15.31	60.44 ± 15.43	51.37 ± 12.95	42.21 ± 11.45	< 0.001
LDL‐C (mg/dL)	116.72 ± 34.96	110.82 ± 31.07	119.59 ± 32.73	119.79 ± 39.76	< 0.001
CTI	8.75 ± 0.85	8.17 ± 0.60	8.68 ± 0.64	9.41 ± 0.79	< 0.001
CVAI	96.23 ± 40.04	54.02 ± 17.75	94.06 ± 12.03	140.63 ± 24.32	< 0.001
TyG	8.68 ± 0.67	8.25 ± 0.46	8.63 ± 0.50	9.17 ± 0.69	< 0.001
TyG–CVAI	849.51 ± 391.31	446.60 ± 149.05	810.24 ± 104.57	1291.67 ± 258.38	< 0.001
CTI–CVAI	860.07 ± 408.58	441.27 ± 146.51	813.68 ± 101.85	1325.23 ± 273.83	< 0.001

*Note:* Data are presented as mean ± standard deviation (SD) for normally distributed variables, median (interquartile range, IQR) for skewed variables, or *n* (%) for categorical variables. *p* values were calculated using one‐way ANOVA, the Kruskal–Wallis test, or chi‐square test as appropriate. HbA1c, glycosylated hemoglobin. TyG, triglyceride–glucose index.

Abbreviations: BMI, body mass index; CRP, C‐reactive protein; CTI, C‐reactive protein–triglyceride–glucose index; CVAI, Chinese visceral adiposity index; FBG, fasting blood glucose; HDL‐C, high‐density lipoprotein cholesterol; LDL‐C, low‐density lipoprotein cholesterol; TC, total cholesterol; WC, waist circumference.

### 3.2. Association of CTI, CVAI, and Their Joint Effect With Incident Stroke

A graded increase in stroke frequency was observed across ascending tertiles of both CTI and CVAI. The event density for CTI escalated from 6.90 per 1000 person‐years in T1 to 15.29 in T3. A parallel trend occurred for CVAI, rising from 6.80 to 17.56 per 1000 person‐years. Kaplan–Meier survival curves (Figure S2) visually confirmed that elevated baseline levels of either index were significantly associated with shortened stroke‐free survival (log‐rank *p* < 0.05). After controlling for confounders in multivariable Cox regression (Table 2, Model 3), participants in T3 compared to those in T1 exhibited a 91% higher risk of incident stroke for CTI (HR: 1.91, 95% CI: 1.56–2.33) and a 96% higher risk for CVAI (HR: 1.96, 95% CI: 1.60–2.38; both *p* < 0.001).

**TABLE 2:** Association of CTI and CVAI and their joint effect with the risk of stroke incidence.

	Model 1		Model 2		Model 3	
	HR (95%CI)	*p*	HR (95%CI)	*p*	HR (95%CI)	*p*
CTI						
Tertile 1	1.00 (Reference)		1.00 (Reference)		1.00 (Reference)	
Tertile 2	1.91 (1.58∼2.31)	< 0.001	1.83 (1.51∼2.21)	< 0.001	1.69 (1.40∼2.05)	< 0.001
Tertile 3	2.29 (1.90∼2.75)	< 0.001	2.22 (1.84∼2.67)	< 0.001	1.91 (1.56∼2.33)	< 0.001
CVAI						
Tertile 1	1.00 (Reference)		1.00 (Reference)		1.00 (Reference)	
Tertile 2	1.56 (1.28∼1.91)	< 0.001	1.53 (1.26∼1.87)	< 0.001	1.41 (1.15∼1.73)	< 0.001
Tertile 3	2.59 (2.16∼3.11)	< 0.001	2.38 (1.97∼2.88)	< 0.001	1.96 (1.60∼2.38)	< 0.001
Joint effect of CTI and CVAI						
Low CTI and low CVAI	1.00 (Reference)		1.00 (Reference)		1.00 (Reference)	
High CTI and low CVAI	1.60 (1.26∼2.03)	< 0.001	1.60 (1.26∼2.03)	< 0.001	1.50 (1.18∼1.92)	0.001
Low CTI amd high CVAI	1.86 (1.48∼2.33)	< 0.001	1.68 (1.34∼2.12)	< 0.001	1.52 (1.20∼1.91)	< 0.001
High CTI and high CVAI	2.52 (2.11∼3.01)	< 0.001	2.39 (1.99∼2.86)	< 0.001	1.98 (1.63∼2.40)	< 0.001

*Note:* Model 1: unadjusted. Model 2: adjusted for age, sex, education, marital status, and residence. Model 3: adjusted for variables in Model 2 plus smoking, drinking, diabetes, hypertension, TC, and LDL‐C.

Abbreviations: CI, confidence interval; CTI, C‐reactive protein–triglyceride–glucose index; CVAI, Chinese visceral adiposity index; HR, hazard ratio; LDL‐C, low‐density lipoprotein cholesterol; TC, total cholesterol.

Evaluating the cumulative impact of these markers through joint categorization revealed that the “double‐high” phenotype (high CTI with high CVAI) possessed the most pronounced risk profile. This group exhibited an incidence rate of 16.71 per 1000 person‐years compared to 6.80 in the “double‐low” reference group. In the fully adjusted model, simultaneous elevation of both markers conferred the highest risk (HR: 1.98, 95% CI: 1.63–2.40, *p* < 0.001). RCS nested within Model 3 demonstrated that the association of continuous CTI and CVAI levels with stroke risk followed a linear trajectory (*p* for nonlinearity > 0.05; Figure 1A,B). No significant biological interaction was found on either additive or multiplicative scales (Table S4).

**FIGURE 1:**
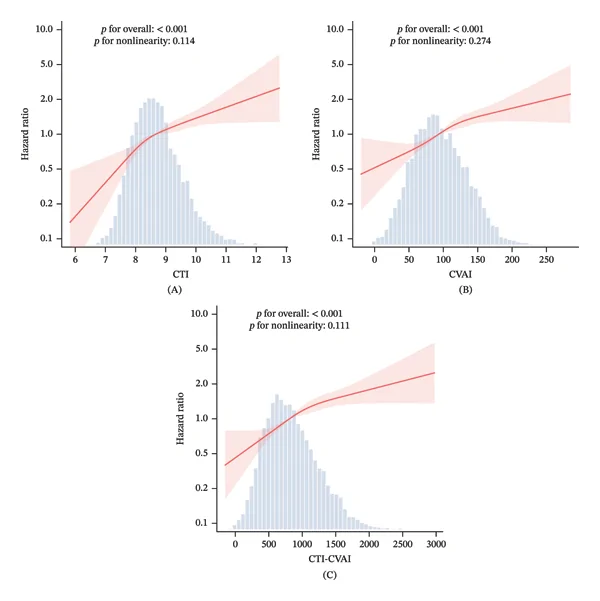
Nonlinear associations of CTI, CVAI, and CTI–CVAI with the risk of incident stroke. The graphs demonstrate the hazard ratios (HRs) and 95% confidence intervals (CIs) derived from Cox proportional hazards regression Model 3. (A) CTI; (B) CVAI; (C) CTI–CVAI. The models were adjusted for sex, education, marital status, residence, smoking status, alcohol consumption, diabetes, hypertension, age, TC, and LDL‐C. Solid lines represent the estimated HRs, and shaded areas represent the 95% CIs.

### 3.3. Construction and Performance of the Novel CTI–CVAI Index

The derived CTI–CVAI composite metric exhibited a pronounced, dose‐dependent relationship with stroke onset. Incidence density escalated progressively from 6.41 per 1000 person‐years (95% CI: 5.43–7.51) in the bottom tertile to 17.68 (95% CI: 16.00–19.48) in the top tertile. Kaplan–Meier analysis reinforced this finding, linking higher CTI–CVAI scores to reduced stroke‐free survival probability (log‐rank *p* < 0.001; Figure S2D). In the fully adjusted Model 3, participants in T2 and T3 faced significantly higher stroke risks compared to T1, with HRs of 1.53 (95% CI: 1.25–1.88) and 2.14 (95% CI: 1.75–2.62), respectively (Figure 2). A linear dose–response association between continuous CTI–CVAI levels and stroke risk was also established (*p* for nonlinearity = 0.111; Figure 1C).

**FIGURE 2:**
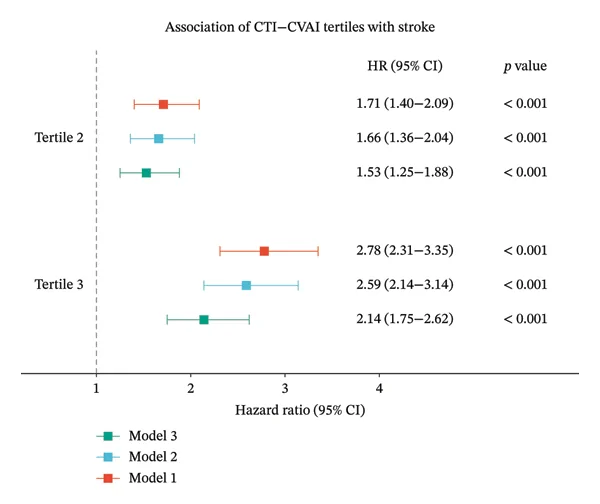
Association between CTI–CVAI tertiles and the risk of incident stroke across different models. The forest plot illustrates the hazard ratios (HRs) and 95% confidence intervals (CIs) for stroke incidence in CTI–CVAI tertiles (with the lowest tertile as the reference). Results are presented for the unadjusted model (Model 1), the partially adjusted model (Model 2), and the fully adjusted model (Model 3). Model 1: unadjusted. Model 2: adjusted for age, sex, education, marital status, and residence. Model 3: adjusted for variables in Model 2 plus smoking, drinking, diabetes, hypertension, TC, and LDL‐C. Abbreviations: HR, hazard ratio; CI, confidence interval; CTI, C‐reactive protein–triglyceride–glucose index; CVAI, Chinese visceral adiposity index; TC, total cholesterol; LDL‐C, low‐density lipoprotein cholesterol.

### 3.4. Predictive Performance of the CTI–CVAI Index

The CTI–CVAI index outperformed all single‐component metrics in forecasting stroke events, attaining the highest AUC value of 0.627 (95% CI: 0.607–0.647; Figure 3A). Pairwise comparison confirmed that CTI–CVAI provided a significant improvement in predictive accuracy over the established TyG–CVAI index (AUC: 0.627 vs. 0.624, *p* = 0.002; Figure 3B). Complementary analysis using the Boruta algorithm identified CTI, TyG, and CVAI as the top three determinants, followed by the composite indices (Figure 3C).

**FIGURE 3:**
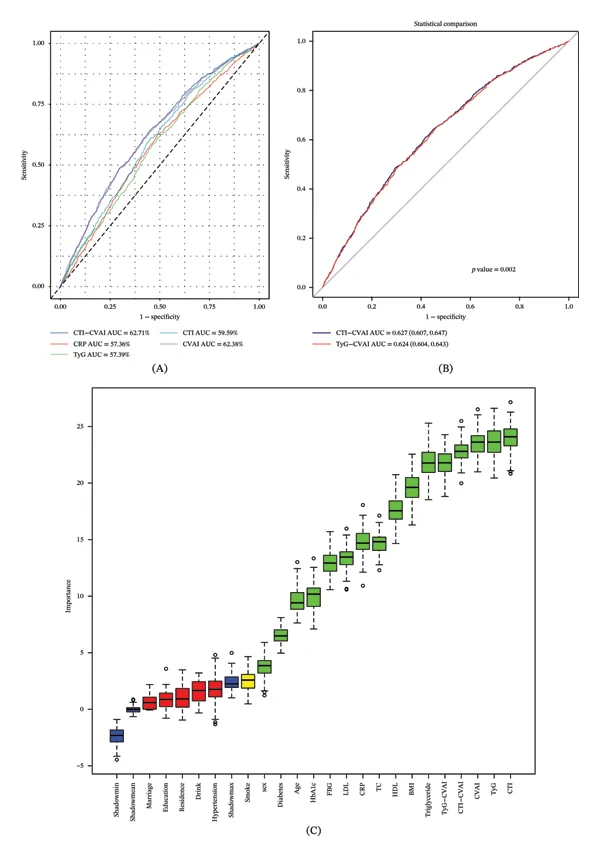
Evaluation of the predictive performance and feature importance of CTI–CVAI for incident stroke. (A) ROC curves comparing the CTI–CVAI index with its individual components (TyG, CRP, CTI, and CVAI). (B) Comparison of ROC curves between the CTI–CVAI index and the TyG–CVAI index. The CTI–CVAI index showed a significantly higher AUC compared to TyG–CVAI (*p* = 0.002). (C) Ranking of variable importance based on the boruta feature selection algorithm. The graph displays the top five predictors (CTI, TyG, CVAI, CTI–CVAI, and TyG–CVAI) ranked in descending order of importance. Abbreviations: ROC, receiver operating characteristic; AUC, area under the curve; BMI, body mass index; CRP, C‐reactive protein; CTI, C‐reactive protein–triglyceride–glucose index; CVAI, Chinese visceral adiposity index; FBG, fasting blood glucose; HbA1c, glycosylated hemoglobin; HDL, high‐density lipoprotein cholesterol; LDL, low‐density lipoprotein cholesterol; TC, total cholesterol; TyG, triglyceride–glucose index.

### 3.5. Subgroup Analyses and Sensitivity Testing

Stratified analyses based on the median CTI–CVAI score (809.94) demonstrated consistent positive associations with stroke risk across most demographic and clinical categories (Table S5). Educational attainment emerged as a significant effect modifier (*p* for interaction = 0.010), with the risk magnitude being more pronounced in participants with higher education (HR: 4.06, 95% CI: 2.07–7.99) compared to those with lower education (HR: 1.54, 95% CI: 1.31–1.80). A comparable interaction was observed between CVAI and education (*p* for interaction = 0.043, Table S7).

Rigorous sensitivity analyses substantiated these primary findings. Excluding events within the first 24 months to mitigate reverse causality yielded consistent results for both the high‐risk combination group (HR = 1.97; Table S8) and the highest tertile (HR = 2.12; Table S9). The associations remained statistically significant after additional adjustments for BMI and therapeutic regimens for hypertension, diabetes, and dyslipidemia (Tables S10–S13). These findings also withstood the stringent Bonferroni correction for multiple testing (Tables S14 and S15). Even within the lag analysis cohort, CTI–CVAI maintained superior predictive accuracy over the TyG–CVAI index (AUC 0.626 vs. 0.623, *p* = 0.002; Figure S3).

## 4. Discussion

Leveraging data from a nationwide longitudinal cohort spanning 8 years, this study comprehensively evaluated the independent and synergistic impact of inflammatory‐metabolic markers on stroke onset. We identified both CTI and CVAI as independent predictors of cerebrovascular events, characterized by distinct linear dose–response trajectories. Joint stratification revealed that individuals with concurrent elevations in both indices faced the steepest risk. The newly derived CTI–CVAI composite metric demonstrated superior prognostic capability, outperforming not only its constituent components but also the conventional TyG–CVAI benchmark. These associations remained robust across numerous sensitivity analyses, establishing the CTI–CVAI index as an effective tool for improving risk assessment in middle‐aged and older Chinese populations.

By integrating metabolic and inflammatory components, the CTI offers a more thorough evaluation of cardiovascular disease risk (CVD) than individual indicators. Our study shows a positive linear association between CTI and incident stroke, which is consistent with previous studies [16, 20, 21]. Huo et al. demonstrated that this relationship persists across different glycemic states, including prediabetes and normoglycemia [20]. Similarly, Yang et al. emphasized the need for maintaining low CTI levels by pointing out that cumulative CTI exposure and longitudinal changes are crucial for stroke risk in older persons [16]. CTI also exhibits better predictive performance than the TyG index or CRP alone, particularly in metabolically heterogeneous populations. For example, Ou et al. discovered that CTI outperformed TyG in predicting cardiovascular outcomes among patients with CKM stages 0–3 [22], likely because glycemic states can confound TyG’s prognostic ability [23]. These findings are supported by our data, which validate CTI as a trustworthy biomarker for stroke risk prediction.

The CVAI has proven to be a reliable measure of visceral fat accumulation, particularly in East Asian populations [24, 25]. Prior research indicates that CVAI is more closely linked to CVD risk than conventional anthropometric indices such as the waist‐to‐hip ratio, visceral adiposity index, and lipid accumulation product [26, 27] and exhibits a linear association with stroke risk [28]. Our findings confirm this linear positive association. While recent studies on the TyG–CVAI score reported a U‐shaped relationship with stroke risk [12]. The potential of combining CTI with CVAI remained unexplored. We discovered that CTI–CVAI is linearly associated with stroke risk and provides better predictive accuracy than TyG–CVAI. This suggests that integrating the inflammatory status captured by CTI into visceral adiposity assessment offers a more refined risk stratification tool than relying on metabolic and adiposity markers alone.

Beyond the composite index, we analyzed the joint effect of CTI and CVAI categories. While one prior study reported that simultaneous elevation of body fat percentage and CTI increased CVD risk by 1.48‐fold [29], our research specifically examines the combined effects of CTI and CVAI. Across all models, individuals with concurrently high CTI and high CVAI faced the highest risk. In the fully adjusted model, this dual‐high phenotype was associated with a 98% increased risk of stroke compared to the double‐low reference group. Although no statistical interaction was observed, these results indicate a strong additive effect, emphasizing that simultaneous assessment of immunometabolic status and visceral fat identifies a high‐risk subpopulation easily overlooked by single‐marker screening.

The mechanism underlying the augmented stroke risk associated with elevated CTI and CVAI is likely multifactorial. The CTI captures both insulin resistance and persistent low‐grade inflammation, which are primary drivers of atherosclerosis and endothelial dysfunction [21]. Evidence suggests CTI remains a stable predictor across various metabolic states [23] and effectively predicts poor prognosis in conditions like cancer cachexia, where TyG is less effective [30]. Concurrently, CVAI measures visceral fat, which is metabolically active and releases proinflammatory cytokines (e.g., TNF‐α and IL‐6) alongside free fatty acids [31, 32]. Regardless of insulin sensitivity, visceral adiposity is independently linked to vascular damage [33]. The interaction of visceral fat gain, systemic inflammation, and insulin resistance accelerates this vascular damage [8]. This synergistic pathophysiology explains why the combined assessment yields the highest predictive efficacy for stroke.

This study has a number of noteworthy advantages. To our knowledge, it is the first prospective cohort research to develop the CTI–CVAI index and validate its superiority over the TyG–CVAI index in stroke prediction. Utilizing a large, nationally representative sample with an extended follow‐up period (> 8 years) provides robust evidence for the synergistic effect of metabolic inflammation and visceral adiposity. Furthermore, the index relies on routine clinical parameters, ensuring its practical utility in primary care. However, several limitations warrant consideration. First, exposure variables were assessed only at baseline, precluding the analysis of dynamic changes or cumulative effects over time. Second, stroke episodes were determined via self‐reports and doctor diagnoses rather than medical records or imaging, potentially introducing recall bias, though this method is standard in large epidemiological surveys. Third, data constraints prevented us from distinguishing between ischemic and hemorrhagic stroke subtypes, which may differ in their pathophysiological associations with the index. Fourth, there is a mathematical overlap of TG in the CTI and CVAI formulas. While this increases the statistical weight of TG, it biologically reflects two distinct mechanisms: hepatic insulin resistance and visceral fat accumulation. Similar to the established TyG–CVAI index, our index remains robust and captures additional inflammatory risks through CRP. Lastly, because CVAI was created specifically for the Chinese population, the CTI–CVAI index requires validation in other ethnic groups.

## 5. Conclusion

In conclusion, elevated CTI and CVAI are independently and linearly associated with an increased risk of incident stroke in middle‐aged and older Chinese adults. Individuals with simultaneous elevations in both indices face the highest risk, demonstrating a strong additive effect. The novel CTI–CVAI index, created by multiplying these two components, exhibited superior predictive performance for stroke compared to either metric alone or the established TyG–CVAI index. By integrating systemic inflammation, insulin resistance, and visceral adiposity using readily available clinical parameters, CTI–CVAI serves as a highly efficient and practical tool for early stroke risk stratification.

## Funding

No funding was received for this manuscript.

## Conflicts of Interest

The authors declare no conflicts of interest.

## Supporting Information

Additional supporting information can be found online in the Supporting Information section.

## Supporting information


**Supporting Information** Figure S1 Flowchart illustrating the selection of the study population. Figure S2 Kaplan–Meier plots of stroke incidence across different groups. (A) Survival curves for CTI tertiles; (B) Survival curves for CVAI tertiles; (C) Survival curves for combined CTI and CVAI groups; (D) Survival curves for CTI–CVAI tertiles. Figure S3 Comparison of ROC curves between CTI–CVAI and TyG–CVAI, excluding early stroke cases. The analysis excluded participants who developed a stroke within 2 years of follow‐up. The CTI–CVAI index maintained superior predictive performance compared to the TyG–CVAI index (AUC: 0.626 vs. 0.623; *p* = 0.002). Table S1 Mathematical formulas for the calculation of metabolic and inflammatory indices. Table S2 Baseline characteristics of participants according to tertiles of the CTI index. Table S3 Baseline characteristics of participants according to tertiles of the CVAI index. Table S4 Interaction between stroke risk and the CTI index and CVAI. Table S5 Subgroup analysis of the relationship between incident stroke and the CTI–CVAI index. Table S6 Subgroup analyses of the association between CTI and incident stroke. Table S7 Subgroup analyses of the association between CVAI and incident stroke. Table S8 Sensitivity analysis of the association between joint CTI and CVAI groups and incident stroke after excluding participants who developed stroke within 2 years of follow‐up. Table S9 Sensitivity analysis of the association between CTI–CVAI tertiles and incident stroke after excluding participants who developed stroke within 2 years of follow‐up. Table S10 Sensitivity analysis of the association between joint CTI and CVAI groups and incident stroke with additional adjustment for medication usage. Table S11 Sensitivity analysis of the association between CTI–CVAI tertiles and incident stroke adjusting for medication usage. Table S12 Sensitivity analysis of the association between joint CTI and CVAI groups and incident stroke after further adjustment for BMI and medication use. Table S13 Sensitivity analysis of the association between CTI–CVAI tertiles and incident stroke with additional adjustment for medication use and BMI. Table S14 Sensitivity analysis: Association of CTI and CVAI and their joint effect with incident stroke using Bonferroni correction for multiple comparisons. Table S15 Sensitivity analysis: Association of CTI–CVAI with incident stroke using Bonferroni correction for multiple comparisons.

## Data Availability

The data that support the findings of this study are available on the CHARLS website at https://charls.pku.edu.cn/.
