# Supplementary material for: Joint Association of Inflammatory‐Metabolic Indices With Risk of Incident Stroke Among Middle‐Aged and Older Chinese Adults
Source: J Obes. 2026 Sep 28;2026:8894079. doi: 10.1155/jobe/8894079 (PMC13618367; doi:10.1155/jobe/8894079)
Supplement: Supplementary file 1 [file JOBE-2026-8894079-s001.zip › Supplementary_2.docx]

**Fig. S1** Flowchart illustrating the selection of the study population.

**Table S1 Mathematical formulas for the calculation of metabolic and inflammatory indices**

| **Index** | **Formula / Calculation Algorithm** | **Notes & Units** |
| --- | --- | --- |
| **TyG Index** | Ln [TG × FBG / 2] | Units: TG and Glucose must be in mg/dL.  If lab data is in mmol/L, convert as follows: TG: 1 mmol/L=88.57 mg/dL  FBG: 1 mmol/L=18.02 mg/dL |
| **CTI** (CRP-TyG Index) | CTI = 0.412 × Ln [CRP] + Ln [TG × FBG/2] | Represents the interaction between insulin resistance and systemic inflammation. |
| **CVAI** (Chinese Visceral Adiposity Index) | **Males:** CVAI = − 267.93 + 0.68 × age + 0.03 × BMI + 4.00 × WC + 22.00 × Log _10_ (TG) − 16.32 × HDL-C  **Females:** CVAI = − 187.32 + 1.71 × age + 4.23 × BMI + 1.12 × WC + 39.76 × Log _10_ (TG) − 11.66 × HDL-C | **Units:**  **Age (years)**  **BMI (kg/m²)**  **WC (cm)**  **TG and HDL-C (mmol/L)** |
| **TyG-CVAI** | TyG Index × CVAI | Composite index of insulin resistance and visceral adiposity. |
| **CTI-CVAI** | CTI × CVAI | **Novel Composite:** Integrates inflammation, insulin resistance, and visceral adiposity. |

**Abbreviations: TyG: Triglyceride-glucose index; CTI: CRP-TyG Index; CVAI: Chinese Visceral Adiposity Index; TG: Triglycerides; BMI: Body Mass Index; WC: Waist Circumference; HDL-C: High-density lipoprotein cholesterol; CRP: C-reactive protein**

**Table S2 Baseline characteristics of participants according to tertiles of the CTI index**

| Characteristics | Total (n=8,267) | Tertile 1 (n=2,756) | Tertile 2 (n=2,755) | Tertile 3 (n=2,756) | *P*-value |
| --- | --- | --- | --- | --- | --- |
| **Age (years)** | 59.40 ± 9.23 | 58.26 ± 9.12 | 60.12 ± 9.35 | 59.81 ± 9.12 | < 0.001 |
| **Sex, n (%)** |  |  |  |  | < 0.001 |
| Female | 4421 (53.5) | 1385 (50.3) | 1483 (53.8) | 1553 (56.3) |  |
| Male | 3846 (46.5) | 1371 (49.7) | 1272 (46.2) | 1203 (43.7) |  |
| **Education, n (%)** |  |  |  |  | 0.504 |
| Below junior high school | 7531 (91.1) | 2503 (90.8) | 2524 (91.6) | 2504 (90.9) |  |
| High school and above | 736 (8.9) | 253 (9.2) | 231 (8.4) | 252 (9.1) |  |
| **Marital status, n (%)** |  |  |  |  | 0.263 |
| Married/Cohabiting | 6910 (83.6) | 2319 (84.1) | 2277 (82.6) | 2314 (84.0) |  |
| Other | 1357 (16.4) | 437 (15.9) | 478 (17.4) | 442 (16.0) |  |
| **Residence, n (%)** |  |  |  |  | < 0.001 |
| Urban | 2695 (32.6) | 767 (27.8) | 870 (31.6) | 1058 (38.4) |  |
| Rural | 5572 (67.4) | 1989 (72.2) | 1885 (68.4) | 1698 (61.6) |  |
| **Smoking, n (%)** | 2512 (30.5) | 899 (32.7) | 838 (30.5) | 775 (28.3) | 0.002 |
| **Drinking, n (%)** | 2723 (33.0) | 1006 (36.5) | 894 (32.5) | 823 (29.9) | < 0.001 |
| **WC (cm)** | 85.13 ± 10.05 | 81.19 ± 8.55 | 84.99 ± 9.71 | 89.21 ± 10.16 | < 0.001 |
| **BMI (kg/m²)** | 23.46 ± 3.86 | 22.17 ± 3.19 | 23.38 ± 3.84 | 24.84 ± 4.04 | < 0.001 |
| **Diabetes, n (%)** | 1250 (15.1) | 136 (4.9) | 303 (11.0) | 811 (29.4) | < 0.001 |
| **Hypertension, n (%)** | 4592 (55.7) | 1208 (44.0) | 1524 (55.5) | 1860 (67.7) | < 0.001 |
| **CRP (mg/L)** | 1.03 (0.55, 2.15) | 0.52 (0.36, 0.82) | 1.07 (0.65, 1.84) | 2.24 (1.22, 4.63) | < 0.001 |
| **FBG (mg/dL)** | 110.28 ± 36.26 | 97.69 ± 14.70 | 104.84 ± 19.53 | 128.29 ± 53.24 | < 0.001 |
| **HbA1c (%)** | 5.27 ± 0.81 | 5.08 ± 0.46 | 5.18 ± 0.55 | 5.55 ± 1.15 | < 0.001 |
| **TC (mg/dL)** | 194.01 ± 38.76 | 184.21 ± 34.18 | 193.52 ± 36.04 | 204.28 ± 42.88 | < 0.001 |
| **HDL-C (mg/dL)** | 51.34 ± 15.31 | 59.24 ± 14.71 | 52.02 ± 13.77 | 42.75 ± 12.66 | < 0.001 |
| **LDL-C (mg/dL)** | 116.72 ± 34.96 | 112.45 ± 30.05 | 120.58 ± 33.27 | 117.14 ± 40.34 | < 0.001 |
| **CTI** | 8.75 ± 0.85 | 7.88 ± 0.33 | 8.67 ± 0.20 | 9.70 ± 0.60 | < 0.001 |
| **CVAI** | 96.23 ± 40.04 | 72.43 ± 30.89 | 96.23 ± 35.58 | 120.05 ± 38.16 | < 0.001 |
| **TyG** | 8.68 ± 0.67 | 8.12 ± 0.34 | 8.62 ± 0.34 | 9.31 ± 0.65 | < 0.001 |
| **TyG-CVAI** | 849.51 ± 391.31 | 590.71 ± 257.50 | 832.21 ± 313.36 | 1125.60 ± 389.11 | < 0.001 |
| **CTI-CVAI** | 860.07 ± 408.58 | 573.30 ± 250.41 | 835.67 ± 312.68 | 1171.21 ± 400.91 | < 0.001 |

**Data are presented as mean ± standard deviation (SD) for normally distributed variables, median (interquartile range, IQR) for skewed variables, or n (%) for categorical variables. P-values were calculated using one-way ANOVA, Kruskal-Wallis test, or Chi-square test as appropriate. Abbreviations: BMI, body mass index; CRP, C-reactive protein; CTI, C-reactive protein-triglyceride-glucose index; CVAI, Chinese visceral adiposity index; FBG, fasting blood glucose; HbA1c, glycosylated hemoglobin; HDL-C, high-density lipoprotein cholesterol; LDL-C, low-density lipoprotein cholesterol; TC, total cholesterol; TyG, triglyceride-glucose index; WC, waist circumference.**

**Table S3 Baseline characteristics of participants according to tertiles of the CVAI index**

| Characteristics | Total (n=8,267) | Tertile 1 (n=2,756) | Tertile 2 (n=2,755) | Tertile 3 (n=2,756) | *P-*value |
| --- | --- | --- | --- | --- | --- |
| **Age (years)** | 59.40 ± 9.23 | 57.36 ± 8.66 | 59.19 ± 9.05 | 61.64 ± 9.47 | < 0.001 |
| **Sex, n (%)** |  |  |  |  | < 0.001 |
| Female | 4421 (53.5) | 1212 (44.0) | 1681 (61.0) | 1528 (55.4) |  |
| Male | 3846 (46.5) | 1544 (56.0) | 1074 (39.0) | 1228 (44.6) |  |
| **Education, n (%)** |  |  |  |  | 0.226 |
| Below junior high school | 7531 (91.1) | 2527 (91.7) | 2513 (91.2) | 2491 (90.4) |  |
| High school and above | 736 (8.9) | 229 (8.3) | 242 (8.8) | 265 (9.6) |  |
| **Marital status, n (%)** |  |  |  |  | 0.693 |
| Married/Cohabiting | 6910 (83.6) | 2317 (84.1) | 2294 (83.3) | 2299 (83.4) |  |
| Other | 1357 (16.4) | 439 (15.9) | 461 (16.7) | 457 (16.6) |  |
| **Residence, n (%)** |  |  |  |  | < 0.001 |
| Urban | 2695 (32.6) | 685 (24.9) | 863 (31.3) | 1147 (41.6) |  |
| Rural | 5572 (67.4) | 2071 (75.1) | 1892 (68.7) | 1609 (58.4) |  |
| **Smoking, n (%)** | 2512 (30.5) | 1116 (40.6) | 708 (25.8) | 688 (25.1) | < 0.001 |
| **Drinking, n (%)** | 2723 (33.0) | 1085 (39.4) | 804 (29.2) | 834 (30.3) | < 0.001 |
| **WC (cm)** | 85.13 ± 10.05 | 75.70 ± 5.44 | 84.39 ± 5.45 | 95.31 ± 7.11 | < 0.001 |
| **BMI (kg/m²)** | 23.46 ± 3.86 | 20.49 ± 2.24 | 23.15 ± 2.49 | 26.75 ± 3.71 | < 0.001 |
| **Diabetes, n (%)** | 1250 (15.1) | 237 (8.6) | 369 (13.4) | 644 (23.4) | < 0.001 |
| **Hypertension, n (%)** | 4592 (55.7) | 1073 (39.1) | 1487 (54.1) | 2032 (73.9) | < 0.001 |
| **CRP (mg/L)** | 1.03 (0.55, 2.15) | 0.72 (0.43, 1.50) | 0.95 (0.53, 1.99) | 1.44 (0.80, 2.83) | < 0.001 |
| **FBG (mg/dL)** | 110.28 ± 36.26 | 103.43 ± 28.65 | 108.67 ± 33.32 | 118.73 ± 43.50 | < 0.001 |
| **HbA1c (%)** | 5.27 ± 0.81 | 5.14 ± 0.65 | 5.25 ± 0.82 | 5.43 ± 0.91 | < 0.001 |
| **TC (mg/dL)** | 194.01 ± 38.76 | 187.19 ± 36.07 | 193.64 ± 37.13 | 201.19 ± 41.60 | < 0.001 |
| **HDL-C (mg/dL)** | 51.34 ± 15.31 | 59.98 ± 15.55 | 51.29 ± 13.40 | 42.75 ± 11.54 | < 0.001 |
| **LDL-C (mg/dL)** | 116.72 ± 34.96 | 111.31 ± 31.64 | 119.21 ± 33.19 | 119.67 ± 39.02 | < 0.001 |
| **CTI** | 8.75 ± 0.85 | 8.27 ± 0.67 | 8.70 ± 0.72 | 9.29 ± 0.84 | < 0.001 |
| **CVAI** | 96.23 ± 40.04 | 53.57 ± 17.18 | 93.80 ± 10.14 | 141.33 ± 23.47 | < 0.001 |
| **TyG** | 8.68 ± 0.67 | 8.31 ± 0.49 | 8.65 ± 0.55 | 9.09 ± 0.71 | < 0.001 |
| **TyG-CVAI** | 849.51 ± 391.31 | 446.84 ± 149.62 | 812.50 ± 109.47 | 1289.17 ± 260.99 | < 0.001 |
| **CTI-CVAI** | 860.07 ± 408.58 | 444.92 ± 151.63 | 817.24 ± 120.01 | 1318.02 ± 281.85 | < 0.001 |

**Data are presented as mean ± standard deviation (SD) for normally distributed variables, median (interquartile range, IQR) for skewed variables, or n (%) for categorical variables. P-values were calculated using one-way ANOVA, Kruskal-Wallis test, or Chi-square test as appropriate. Abbreviations: BMI, body mass index; CRP, C-reactive protein; CTI, C-reactive protein-triglyceride-glucose index; CVAI, Chinese visceral adiposity index; FBG, fasting blood glucose; HbA1c, glycosylated hemoglobin; HDL-C, high-density lipoprotein cholesterol; LDL-C, low-density lipoprotein cholesterol; TC, total cholesterol; TyG, triglyceride-glucose index; WC, waist circumference.**

**Fig. S2** Kaplan–Meier plots of stroke incidence across different groups. (A) Survival curves for CTI tertiles; (B) Survival curves for CVAI tertiles; (C) Survival curves for combined CTI and CVAI groups; (D) Survival curves for CTI-CVAI tertiles.

**Table S4** Interaction between stroke risk and the CTI index and CVAI

| Interactive indices | Interactive effects (95% CI) | | |
| --- | --- | --- | --- |
|  | Model 1 | Model 2 | Model 3 |
| Additive effect |  |  |  |
| RERI | 0.07 (- 0.45 ~ 0.59) | 0.11 (- 0.38 ~ 0.61) | - 0.04 (- 0.5 ~ 0.43) |
| AP | 0.03 (- 0.18 ~ 0.23) | 0.05 (- 0.16 ~ 0.25) | - 0.02 (-0.25 ~ 0.22) |
| SI | 1.05 (0.73 ~ 1.49) | 1.09 (0.74 ~ 1.6) | 0.97 (0.61 ~ 1.52) |
| Multiplicative effect | 0.85 (0.62 ~ 1.16) | 0.89 (0.65 ~ 1.21) | 0.87 (0.64 ~ 1.19) |

Model 1: Unadjusted. Model 2: Adjusted for age, sex, education, marital status, and residence. Model 3: Adjusted for variables in Model 2 plus smoking, drinking, diabetes, hypertension, TC, and LDL-C. Abbreviations: AP, Attributable Proportion due to interaction; CI, Confidence Interval; CTI, C-reactive protein-triglyceride-glucose index; CVAI, Chinese visceral adiposity index; HR, Hazard Ratio; LDL-C, low-density lipoprotein cholesterol; RERI, Relative Excess Risk due to Interaction; SI, Synergy Index; TC, total cholesterol.

**Table S5** Subgroup analysis of the relationship between incident stroke and the CTI-CVAI index

| Subgroup | No. of Cases / Total | HR (95% CI) for High vs. Low CTI-CVAI | *P-*value | *P* for Interaction |
| --- | --- | --- | --- | --- |
| Age, years |  |  |  | 0.430 |
| < 60 | 354 / 4,501 | 1.68 (1.33–2.11) | < 0.001 |  |
| ≥ 60 | 462 / 3,766 | 1.59 (1.29–1.97) | < 0.001 |  |
| Sex |  |  |  | 0.071 |
| Female | 427 / 4,421 | 1.45 (1.16–1.80) | 0.001 |  |
| Male | 389 / 3,846 | 1.86 (1.49–2.32) | < 0.001 |  |
| Education |  |  |  | 0.010 |
| Lower than junior high | 749 / 7,531 | 1.54 (1.31–1.80) | < 0.001 |  |
| High school and above | 67 / 736 | 4.06 (2.07–7.99) | < 0.001 |  |
| Marital Status |  |  |  | 0.420 |
| Married/Cohabiting | 666 / 6,910 | 1.69 (1.42–2.01) | < 0.001 |  |
| Other | 150 / 1,357 | 1.43 (0.99–2.05) | 0.054 |  |
| Residence |  |  |  | 0.190 |
| Urban | 283 / 2,695 | 1.89 (1.40–2.54) | < 0.001 |  |
| Rural | 533 / 5,572 | 1.55 (1.29–1.87) | < 0.001 |  |
| Smoking |  |  |  | 0.810 |
| No | 559 / 5,731 | 1.60 (1.32–1.95) | < 0.001 |  |
| Yes | 256 / 2,512 | 1.73 (1.33–2.26) | < 0.001 |  |
| Drinking |  |  |  | 0.456 |
| No | 552 / 5,539 | 1.54 (1.27–1.87) | < 0.001 |  |
| Yes | 264 / 2,723 | 1.88 (1.45–2.45) | < 0.001 |  |
| Diabetes |  |  |  | 0.618 |
| No | 649 / 7,017 | 1.63 (1.38–1.92) | < 0.001 |  |
| Yes | 167 / 1,250 | 1.71 (1.11–2.63) | 0.014 |  |
| Hypertension |  |  |  | 0.372 |
| No | 222 / 3,648 | 1.48 (1.12–1.95) | 0.005 |  |
| Yes | 592 / 4,592 | 1.73 (1.43–2.09) | < 0.001 |  |

Hazard ratios (HRs) were derived from the fully adjusted model (Model 3), which controlled for sex, education, marital status, residence, smoking, drinking, diabetes, hypertension, age, TC, and LDL-C, unless the variable was used for stratification. Abbreviations: CI, Confidence Interval; CTI, C-reactive protein-triglyceride-glucose index; CVAI, Chinese visceral adiposity index; HR, Hazard Ratio; LDL-C, low-density lipoprotein cholesterol; TC, total cholesterol.

**Table S6 Subgroup analyses of the association between CTI and incident stroke**

| Subgroup | No. of Cases / Total | HR (95% CI) for High vs. Low CTI | *P*-value | *P* for Interaction |
| --- | --- | --- | --- | --- |
| **Age, years** |  |  |  | 0.332 |
| < 60 | 354 / 4,501 | 1.61 (1.28–2.03) | < 0.001 |  |
| ≥ 60 | 462 / 3,766 | 1.49 (1.22–1.82) | < 0.001 |  |
| **Sex** |  |  |  | 0.914 |
| Female | 427 / 4,421 | 1.60 (1.29–1.98) | < 0.001 |  |
| Male | 389 / 3,846 | 1.49 (1.20–1.85) | < 0.001 |  |
| **Education** |  |  |  | 0.522 |
| Lower than junior high | 749 / 7,531 | 1.53 (1.30–1.79) | < 0.001 |  |
| High school and above | 67 / 736 | 1.76 (1.02–3.02) | 0.041 |  |
| **Marital Status** |  |  |  | 0.798 |
| Married/Cohabiting | 666 / 6,910 | 1.51 (1.28–1.79) | < 0.001 |  |
| Other | 150 / 1,357 | 1.69 (1.19–2.40) | 0.004 |  |
| **Residence** |  |  |  | 0.538 |
| Urban | 283 / 2,695 | 1.59 (1.22–2.09) | < 0.001 |  |
| Rural | 533 / 5,572 | 1.53 (1.27–1.83) | < 0.001 |  |
| **Smoking** |  |  |  | 0.526 |
| No | 559 / 5,731 | 1.59 (1.32–1.91) | < 0.001 |  |
| Yes | 256 / 2,512 | 1.46 (1.12–1.89) | 0.005 |  |
| **Drinking** |  |  |  | 0.186 |
| No | 552 / 5,539 | 1.39 (1.16–1.67) | < 0.001 |  |
| Yes | 264 / 2,723 | 1.89 (1.45–2.46) | < 0.001 |  |
| **Diabetes** |  |  |  | 0.369 |
| No | 649 / 7,017 | 1.53 (1.31–1.80) | < 0.001 |  |
| Yes | 167 / 1,250 | 1.75 (1.11–2.75) | 0.015 |  |
| **Hypertension** |  |  |  | 0.175 |
| No | 222 / 3,648 | 1.34 (1.01–1.76) | 0.040 |  |
| Yes | 592 / 4,592 | 1.65 (1.38–1.99) | < 0.001 |  |

**Hazard ratios (HRs) were derived from the fully adjusted model (Model 3), which controlled for sex, education, marital status, residence, smoking, drinking, diabetes, hypertension, age, TC, and LDL-C, unless the variable was used for stratification. Abbreviations: CI, Confidence Interval; CTI, C-reactive protein-triglyceride-glucose index; CVAI, Chinese visceral adiposity index; HR, Hazard Ratio; LDL-C, low-density lipoprotein cholesterol; TC, total cholesterol.**

**Table S7 Subgroup analyses of the association between CVAI and incident stroke**

| Subgroup | No. of Cases / Total | HR (95% CI) for High vs. Low CVAI | *P*-value | *P* for Interaction |
| --- | --- | --- | --- | --- |
| **Age, years** |  |  |  | 0.278 |
| < 60 | 354 / 4,501 | 1.66 (1.33–2.09) | < 0.001 |  |
| ≥ 60 | 462 / 3,766 | 1.50 (1.21–1.85) | < 0.001 |  |
| **Sex** |  |  |  | 0.303 |
| Female | 427 / 4,421 | 1.48 (1.19–1.85) | < 0.001 |  |
| Male | 389 / 3,846 | 1.68 (1.35–2.09) | < 0.001 |  |
| **Education** |  |  |  | 0.043 |
| Lower than junior high | 749 / 7,531 | 1.50 (1.28–1.77) | < 0.001 |  |
| High school and above | 67 / 736 | 3.12 (1.65–5.89) | < 0.001 |  |
| **Marital Status** |  |  |  | 0.261 |
| Married/Cohabiting | 666 / 6,910 | 1.66 (1.39–1.97) | < 0.001 |  |
| Other | 150 / 1,357 | 1.30 (0.91–1.87) | 0.151 |  |
| **Residence** |  |  |  | 0.097 |
| Urban | 283 / 2,695 | 1.91 (1.42–2.57) | < 0.001 |  |
| Rural | 533 / 5,572 | 1.47 (1.22–1.76) | < 0.001 |  |
| **Smoking** |  |  |  | 0.757 |
| No | 559 / 5,731 | 1.54 (1.27–1.87) | < 0.001 |  |
| Yes | 256 / 2,512 | 1.67 (1.29–2.17) | < 0.001 |  |
| **Drinking** |  |  |  | 0.508 |
| No | 552 / 5,539 | 1.50 (1.24–1.81) | < 0.001 |  |
| Yes | 264 / 2,723 | 1.78 (1.37–2.32) | < 0.001 |  |
| **Diabetes** |  |  |  | 0.832 |
| No | 649 / 7,017 | 1.58 (1.33–1.87) | < 0.001 |  |
| Yes | 167 / 1,250 | 1.57 (1.05–2.34) | 0.028 |  |
| **Hypertension** |  |  |  | 0.734 |
| No | 222 / 3,648 | 1.52 (1.15–1.99) | 0.003 |  |
| Yes | 592 / 4,592 | 1.62 (1.34–1.96) | < 0.001 |  |

**Hazard ratios (HRs) were derived from the fully adjusted model (Model 3), which controlled for sex, education, marital status, residence, smoking, drinking, diabetes, hypertension, age, TC, and LDL-C, unless the variable was used for stratification. Abbreviations: CI, Confidence Interval; CTI, C-reactive protein-triglyceride-glucose index; CVAI, Chinese visceral adiposity index; HR, Hazard Ratio; LDL-C, low-density lipoprotein cholesterol; TC, total cholesterol.**

**Table S8 Sensitivity analysis of the association between joint CTI and CVAI groups and incident stroke after excluding participants who developed stroke within 2 years of follow-up**

| **Groups** | **HR (95% CI)** | ***P-*value** |
| --- | --- | --- |
| **Low CTI & Low CVAI** | 1.00 (Reference) |  |
| **High CTI & Low CVAI** | 1.50 (1.17–1.91) | 0.001 |
| **Low CTI & High CVAI** | 1.53 (1.21–1.93) | < 0.001 |
| **High CTI & High CVAI** | 1.97 (1.62–2.40) | < 0.001 |

**Abbreviations: HR**: Hazard Ratio; **CI**: Confidence Interval; **TC**: Total Cholesterol; **LDL-C**: Low-Density Lipoprotein Cholesterol. **Model adjusted for:** age, sex, education, marital status, residence, smoking, drinking, diabetes, hypertension, TC, and LDL-C.

**Table S9 Sensitivity analysis of the association between CTI-CVAI tertiles and incident stroke after excluding participants who developed stroke within 2 years of follow-up**

| **Groups** | **HR (95% CI)** | ***P-*value** |
| --- | --- | --- |
| **CTI-CVAI Tertiles** |  |  |
| Tertile 1 | 1.00 (Reference) |  |
| Tertile 2 | 1.52 (1.23–1.87) | < 0.001 |
| Tertile 3 | 2.12 (1.73–2.60) | < 0.001 |

**Abbreviations: HR**: Hazard Ratio; **CI**: Confidence Interval; **TC**: Total Cholesterol; **LDL-C**: Low-Density Lipoprotein Cholesterol. **Model adjusted for:** age, sex, education, marital status, residence, smoking, drinking, diabetes, hypertension, TC, and LDL-C.

**Fig. S3 Comparison of ROC curves between CTI-CVAI and TyG-CVAI excluding early stroke cases. The analysis excluded participants who developed stroke within two years of follow-up. The CTI-CVAI index maintained superior predictive performance compared to the TyG-CVAI index (AUC: 0.626 vs. 0.623; P = 0.002).**

**Table S10 Sensitivity analysis of the association between joint CTI and CVAI groups and incident stroke with additional adjustment for medication usage**

| **Groups** | **HR (95% CI)** | ***P*-value** |
| --- | --- | --- |
| **Low CTI & Low CVAI** | 1.00 (Reference) |  |
| **High CTI & Low CVAI** | 1.50 (1.17–1.91) | 0.001 |
| **Low CTI & High CVAI** | 1.43 (1.13–1.82) | 0.003 |
| **High CTI & High CVAI** | 1.82 (1.50–2.22) | < 0.001 |

**Abbreviations: HR**: Hazard Ratio; **CI**: Confidence Interval; **TC**: Total Cholesterol; **LDL-C**: Low-Density Lipoprotein Cholesterol. **Model adjusted for:** Covariates in Model 3 (age, sex, education, marital status, residence, smoking, drinking, diabetes, hypertension, TC, and LDL-C) plus **antihypertensive drugs, antidiabetic drugs, and lipid-lowering drugs**.

**Table S11 Sensitivity analysis of the association between CTI-CVAI tertiles and incident stroke adjusting for medication usage**

| **Groups** | **HR (95% CI)** | ***P-*value** |
| --- | --- | --- |
| **CTI-CVAI Tertiles** |  |  |
| Tertile 1 | 1.00 (Reference) |  |
| Tertile 2 | 1.51 (1.23–1.86) | < 0.001 |
| Tertile 3 | 1.93 (1.56–2.37) | < 0.001 |

**Abbreviations: HR**: Hazard Ratio; **CI**: Confidence Interval; **TC**: Total Cholesterol; **LDL-C**: Low-Density Lipoprotein Cholesterol. **Model adjusted for:** Covariates in Model 3 (age, sex, education, marital status, residence, smoking, drinking, diabetes, hypertension, TC, and LDL-C) plus **antihypertensive drugs, antidiabetic drugs, and lipid-lowering drugs**.

**Table S12 Sensitivity analysis of the association between joint CTI and CVAI groups and incident stroke after further adjustment for BMI and medication use**

| **Groups** | **HR (95% CI)** | ***P-*value** |
| --- | --- | --- |
| **Low CTI & Low CVAI** | 1.00 (Reference) |  |
| **High CTI & Low CVAI** | 1.50 (1.17–1.92) | 0.001 |
| **Low CTI & High CVAI** | 1.36 (1.06–1.75) | 0.017 |
| **High CTI & High CVAI** | 1.71 (1.37–2.14) | < 0.001 |

**Abbreviations: HR**: Hazard Ratio; **CI**: Confidence Interval; **BMI**: Body Mass Index; **TC**: Total Cholesterol; **LDL-C**: Low-Density Lipoprotein Cholesterol. **Model adjusted for:** covariates in Model 3 (age, sex, education, marital status, residence, smoking, drinking, diabetes, hypertension, TC, LDL-C) **plus BMI and use of antidiabetic, antihypertensive, and lipid-lowering medications.**

**Table S13 Sensitivity analysis of the association between CTI-CVAI tertiles and incident stroke with additional adjustment for medication use and BMI**

| **Groups** | **HR (95% CI)** | ***P*-value** |
| --- | --- | --- |
| **CTI-CVAI Tertiles** |  |  |
| Tertile 1 | 1.00 (Reference) |  |
| Tertile 2 | 1.51 (1.22–1.87) | < 0.001 |
| Tertile 3 | 1.92 (1.49–2.48) | < 0.001 |

**Abbreviations: HR**: Hazard Ratio; **CI**: Confidence Interval; **BMI**: Body Mass Index; **TC**: Total Cholesterol; **LDL-C**: Low-Density Lipoprotein Cholesterol. **Model adjusted for:** covariates in Model 3 (age, sex, education, marital status, residence, smoking, drinking, diabetes, hypertension, TC, LDL-C) **plus BMI and use of antidiabetic, antihypertensive, and lipid-lowering medications.**

**Table S14 Sensitivity analysis: Association of CTI, CVAI and their joint effect with incident stroke using Bonferroni correction for multiple comparisons.**

| **Groups** | Model 1 | **Adjusted *P*** | Model 2 | **Adjusted *P*** | Model 3 | **Adjusted *P*** |
| --- | --- | --- | --- | --- | --- | --- |
|  | **HR (95% CI)** |  | **HR (95% CI)** |  | **HR (95% CI)** |  |
| **CTI** |  |  |  |  |  |  |
| Tertile 1 | 1.00 (Reference) |  | 1.00 (Reference) |  | 1.00 (Reference) |  |
| Tertile 2 | 1.91 (1.58 ~ 2.31) | < 0.021 | 1.83 (1.51 ~ 2.21) | < 0.021 | 1.69 (1.40 ~ 2.05) | < 0.021 |
| Tertile 3 | 2.29 (1.90 ~ 2.75) | < 0.021 | 2.22 (1.84 ~ 2.67) | < 0.021 | 1.91 (1.56 ~ 2.33) | < 0.021 |
| **CVAI** |  |  |  |  |  |  |
| Tertile 1 | 1.00 (Reference) |  | 1.00 (Reference) |  | 1.00 (Reference) |  |
| Tertile 2 | 1.56 (1.28 ~ 1.91) | < 0.021 | 1.53 (1.26 ~ 1.87) | < 0.021 | 1.41 (1.15 ~ 1.73) | < 0.021 |
| Tertile 3 | 2.59 (2.16 ~ 3.11) | < 0.021 | 2.38 (1.97 ~ 2.88) | < 0.021 | 1.96 (1.60 ~ 2.38) | < 0.021 |
| **Joint effect of CTI and CVAI** |  |  |  |  |  |  |
| Low CTI & low CVAI | 1.00 (Reference) |  | 1.00 (Reference) |  | 1.00 (Reference) |  |
| High CTI & low CVAI | 1.60 (1.26 ~ 2.03) | < 0.021 | 1.60 (1.26 ~ 2.03) | < 0.021 | 1.50 (1.18 ~ 1.92) | 0.021 |
| Low CTI & high CVAI | 1.86 (1.48 ~ 2.33) | < 0.021 | 1.68 (1.34 ~ 2.12) | < 0.021 | 1.52 (1.20 ~ 1.91) | < 0.021 |
| High CTI & high CVAI | 2.52 (2.11 ~ 3.01) | < 0.021 | 2.39 (1.99 ~ 2.86) | < 0.021 | 1.98 (1.63 ~ 2.40) | < 0.021 |

Abbreviations: HR: Hazard Ratio; CI: Confidence Interval; BMI: Body Mass Index; TC: Total Cholesterol; LDL-C: Low-Density Lipoprotein Cholesterol. Model adjusted for: covariates in Model 3 (age, sex, education, marital status, residence, smoking, drinking, diabetes, hypertension, TC, LDL-C) plus BMI and use of antidiabetic, antihypertensive, and lipid-lowering medications.

**Table S15** Sensitivity analysis: Association of CTI-CVAI with incident stroke using Bonferroni correction for multiple comparisons.

| **Groups** | Model 1 | | | Model 2 | | | Model 3 | |
| --- | --- | --- | --- | --- | --- | --- | --- | --- |
|  | **HR (95% CI)** | **Adjusted P** | **HR (95% CI)** | | **Adjusted P** | **HR (95% CI)** | | **Adjusted P** |
| **CTI-CVAI** |  |  |  | |  |  | |  |
| Tertile 1 | 1.00 (Reference) |  | 1.00 (Reference) | |  | 1.00 (Reference) | |  |
| Tertile 2 | 1.71 (1.40 ~ 2.09) | < 0.006 | 1.66 (1.36 ~ 2.04) | | < 0.006 | 1.53 (1.25 ~ 1.88) | | < 0.006 |
| Tertile 3 | 2.78 (2.31 ~ 3.35) | < 0.006 | 2.59 (2.14 ~ 3.14) | | < 0.006 | 2.14 (1.75 ~ 2.62) | | < 0.006 |

Abbreviations: HR: Hazard Ratio; CI: Confidence Interval; BMI: Body Mass Index; TC: Total Cholesterol; LDL-C: Low-Density Lipoprotein Cholesterol. Model adjusted for: covariates in Model 3 (age, sex, education, marital status, residence, smoking, drinking, diabetes, hypertension, TC, LDL-C) plus BMI and use of antidiabetic, antihypertensive, and lipid-lowering medications.
